# Smear microscopy and culture conversion rates among smear positive pulmonary tuberculosis patients by HIV status in Dar es Salaam, Tanzania

**DOI:** 10.1186/1471-2334-10-210

**Published:** 2010-07-16

**Authors:** Mbazi Senkoro, Sayoki G Mfinanga, Odd Mørkve

**Affiliations:** 1 National Institute for Medical Research, Muhimbili Medical Research Centre, Dar es Salaam, Tanzania; 2 Centre for International Health, University of Bergen, Bergen, Norway

## Abstract

**Background:**

Tanzania ranks 15^th ^among the world's 22 countries with the largest tuberculosis burden and tuberculosis has continued to be among the major public health problems in the country. Limited data, especially in patients co infected with HIV, are available to predict the duration of time required for a smear positive pulmonary tuberculosis patient to achieve sputum conversion after starting effective treatment. In this study we assessed the sputum smear and culture conversion rates among HIV positive and HIV negative smear positive pulmonary tuberculosis patients in Dar es Salaam

**Methods:**

The study was a prospective cohort study which lasted for nine months, from April to December 2008

**Results:**

A total of 502 smear positive pulmonary tuberculosis patients were recruited. HIV test results were obtained for 498 patients, of which 33.7% were HIV positive.

After two weeks of treatment the conversion rate by standard sputum microscopy was higher in HIV positive(72.8%) than HIV negative(63.3%) patients by univariate analysis(P = 0.046), but not in multivariate analysis. Also after two weeks of treatment the conversion rate by fluorescence microscopy was higher in HIV positive (72.8%) than in HIV negative(63.2%) patients by univariate analysis (P = 0.043) but not in the multivariate analysis. The conversion rates by both methods during the rest of the treatment period (8, 12, and 20 weeks) were not significantly different between HIV positive and HIV negative patients.

With regards to culture, the conversion rate during the whole period of the treatment (2, 8, 12 and 20 weeks) were not significantly different between HIV positive and HIV negative patients.

**Conclusion:**

Conversion rates of standard smear microscopy, fluorescence microscopy and culture did not differ between HIV positive and HIV negative pulmonary tuberculosis patients.

## Background

The World Health Organization (WHO) estimates that there are almost 13.7milion people living with tuberculosis and that the disease kills more young people and adults than any other infectious disease in the world. A total of about 1.77 million people died of tuberculosis in 2007 including 456,000 patients infected with human immunodeficiency virus (HIV). Among the world's 22 countries with the largest tuberculosis burden, Tanzania ranks 15^th ^[1] and tuberculosis has continued to be among the major public health problems in the country[2]. Tuberculosis control aims to reduce the spread of the infection and the most efficient method for preventing transmission is identification and cure of infectious pulmonary tuberculosis patients [3]. Monitoring tuberculosis patients during treatment is vital in order to establish patients' treatment outcomes and to measure the national programme effectiveness [4]. Bacteriological monitoring during treatment is needed in sputum smear positive cases and, also, the microscopy results from specimens collected at the end of treatment are used to confirm the cure of the patients [2]. After conversion to a negative smear the patient is unlikely to transmit tuberculosis to contacts [2,5,6], therefore the conversion rate may be used to determine the period whereby the patient remains potentially infectious [6].

Limited data, especially in patients co-infected with HIV, are available to predict the duration of time required for a smear positive pulmonary tuberculosis patient to achieve sputum conversion after starting effective treatment [7]. This study assessed the sputum smear and culture conversion rates among HIV positive and HIV negative smear positive pulmonary tuberculosis patients in Dar es Salaam.

## Methods

### Study setting, design and population

The study was conducted in Dar es Salaam at Amana, Temeke and Mwananyamala District Hospitals, and Tandale, Magomeni and Mnazi Mmoja Health Centres. The study was a prospective cohort study which lasted for nine months, from April to December 2008. The study subjects were patients attending the selected health facilities, and who were diagnosed, by standard smear microscopy, to have smear positive pulmonary tuberculosis at baseline and who agreed to participate in the study. Patients who did not stay in the study area for the period of follow up and children below the age of 15 years were not included in the study

### Sample size

Allowing 10% drop out, the sample size required was calculated to 500, assuming that the proportion who were still smear positive after 2 months of treatment was 12% and after 5 months 9%[8].

### Data collection

Patient recruitment was done by the District Tuberculosis and Leprosy Coordinators (DTLCs) of the respective health facilities. Recruitment was continuous throughout the study period. All new smear positive pulmonary tuberculosis patients attending Mwananyamala, Amana and Temeke District Hospitals, and Tandale, Magomeni and Mnazi Mmoja health centres, were counseled about the study. After obtaining informed consent, the patients were interviewed and the information obtained, including past history of anti-tuberculosis drugs for prophylactic and/or treatment of active tuberculosis and demographic characteristics such as age, sex, marital status, occupation and level of education, was recorded in questionnaires. After being interviewed the patients were asked to provide one sputum sample for smear microscopy and culture. The sputum collected, together with the two sputa samples from the laboratory which were used for the diagnosis, were sent to the Central Tuberculosis Reference Laboratory (CTRL) for repetition of standard smear microscopy, fluorescence microscopy and culture using Lowenstein-Jensen solid medium.

Patients were treated under Direct Observed Treatment Short Course (DOTS) strategy. The patients were given standard short course treatment of tuberculosis regardless of their HIV status. HIV status of the subjects was identified through the routine National Tuberculosis and Leprosy Program (NTLP) screening of tuberculosis patients. We did not acquire information as to whether the HIV positive patients were on Anti-Retroviral Therapy (ART) or not. Moreover, we did not establish the levels of CD4+count of those patients who were HIV positive or if there was any variation of the level of CD4+counts with time during the follow-up period

All the enrolled patients were seen again after two weeks of treatment. At this visit, another sputum sample was collected and sent to CTRL for culture, standard smear microscopy and fluorescence microscopy to assess conversion to a negative test. Patients who had a persistent positive microscopy and/or culture were followed up again at the 8^th ^week with the same testing as by the end of week 2.

Those who were still positive by microscopy and/or culture were then followed up again at the 12^th ^week, and if they had not converted to negative bacteriology they were followed up again at the 20^th ^week, using the same tests. At the CTRL there is an existing quality control program which is under the NTLP

### Statistical analysis

Completed questionnaires were double entered into a computer software program (Epi Data version 3.1) followed by data cleaning. The data was transferred to SPSS version 15 for analysis. For categorical variables, Pearson Chi-squares and Wald statistics were used, and Student's test was used for continuous variables. The level of significance was set at p ≤ 0.05.

Smear microscopy results were used to estimate the proportion of smear negative patients at 2, 8, 12 and 20 weeks. Culture results were used to estimate the proportion of culture negative patients at 2, 8, 12 and 20 weeks. Logistic regression analysis was used to assess and adjust for potential confounders associated with sputum smear conversion. Candidate independent variables included sex, HIV status, age, past history of using anti TB and education level. The procedure used was backward elimination stepwise method that was based on the probability of the likelihood-ratio statistic. Where appropriate, adjusted odds ratios with 95% confidence intervals are reported.

### Ethical consideration

Ethical clearance was obtained from the Medical Research Coordinating Committee of the Ministry of Health and Social Welfare Tanzania (MoHSW). Permission to conduct the study was sought from the respective authorities where the study was conducted. The goal and benefits of the study were explained to the study participants and oral informed consents were obtained from the participants prior to enrolment.

## Results

A total of 502 smear positive pulmonary tuberculosis patients were recruited. HIV test results were obtained for 498 patients, of which 33.7% were HIV positive. The proportion of males in those who were HIV positive was 64.9% and 71.5% in those who were HIV negative. The mean age was 35.7 ± 9.5 years for HIV positive and 30.1 ± 10.2 years for HIV negative patients. Among HIV positive tuberculosis patients there were more patients within the age group >35 years (47%) as compared to HIV negative tuberculosis patients (22.4%) (X^2 ^= 31.652, d.f. = 1, P = 0.001). With regards to marital status, there were significantly less single (Wald statistic = 12.283, P = 0.01) and cohabiting (Wald statistic = 3.879, P = 0.049), and more married (Wald statistic = 6.320, P = 0.012) individuals between tuberculosis patients who were HIV positive compared to those who were HIV negative. (Table 1)

**Table 1:** General characteristics of smear positive pulmonary tuberculosis patients in Dar es Salaam

Characteristic	HIV Positive N = 168 % (n)	HIV Negative N = 330 % (n)	p-value
**HIV result s**	33.7 (168)	66.3 (330)	
			
**Sex**			
Male	64.9 (109)	71.5 (236)	0.13
Female	35.1 (59)	28.5 (94)	
			
**Mean ± SD age in years**	35.7 ± 9.5	30.1 ± 10.2	0.001
			
**Age groups(years)**			
15-35	53.0 (89)	77.6 (256)	<0.001
>35	47.0 (79)	22.4 (74)	
			
**Age group(years)**			
15-25	11.9(20)	37.6 (124)	<0.001
26-35	41.1 (69)	40.0 (132)	
36-45	33.3 (56)	15.5 (51)	
>45	13.7 (23)	7.0(23)	
			
**Education**			
No education	25.0 (42)	24.2 (80)	0.85
Have education	75.0 (126)	75.8 (250)	
			
**Education level**			
No formal education	8.9(15)	7.6(26)	0.92
Primary education	76.2 (128)	76.7 (253)	
Secondary education and above	14.9 (25)	15.5 (51)	
			
**Occupation**			
Unemployed	27.4 (46)	27.0 (89)	0.79
Employed	15.5 (26)	17.9 (59)	
Self employed	57.1 (96)	55.2 (182)	
			
**Marital status**			
Single	33.3 (56)	51.5 (170)	0.01
Married	41.1 (69)	38.8 (128)	0.012
Cohabiting	3.0(5)	3.3(11)	0.049
Separate/Divorce	14.9 (25)	4.2(14)	0.95
Widowed	7.7(13)	2.1(7)	0.19
			
**BCG scar**			
Present	64.3 (108)	70.3 (232)	0.17
Absent	35.7 (60)	29.7 (98)	

The proportion of patients with past history of using anti-tuberculosis drugs was significantly higher in HIV positive (23.2%) as compared to HIV negative tuberculosis patients (10.9%) (X^2^= 13.177, d.f. = 1, P = 0.001) (Table 2)

**Table 2:** Symptoms and disease past history of smear positive pulmonary tuberculosis patients

Characteristic	HIV Positive N = 168 % (n)	HIV Negative N = 330 % (n)	p-value
**Cough**			
Yes	98.2 (165)	98.5 (325)	0.82
No	1.8(3)	1.5(5)	
			
**Chest pain**			
Yes	69.6 (117)	72.7 (240)	0.47
No	30.4 (51)	27.3 (90)	
			
**Fever**			
Yes	73.8 (124)	68.2(225)	0.20
No	26.2 (44)	31.8(105)	
			
**Night sweats**			
Yes	61.9 (104)	68.2 (225)	0.16
No	38.1 (64)	31.8 (105)	
			
**Loss of weight**			
Yes	88.1 (148)	85.8 (283)	0.47
No	11.9 (20)	14.2 (47)	
			
**Past history of TB**			
Yes	13.7 (23)	8.5(28)	0.07
No	86.3 (145)	91.5 (302)	
			
**Past history of anti-TB**			
Yes	23.2 (39)	10.9 (36)	< 0.001
No	76.8 (129)	89.1 (294)	
			
**Family member with TB**			
Yes	16.1 (27)	18.2 (60)	0.56
No	83.9 (141)	81.8 (270)	

With regards to standard sputum microscopy, after two weeks of treatment the conversion rate was higher in HIV positive (72.8%) than HIV negative(63.3%) patients by univariate analysis, and the difference was statistically significant (X^2^= 3.99, d.f = 1, P = 0.046). However, when we entered the data in the logistic model and did a backward elimination using stepwise method, variables remaining in the model were sex and HIV status. Males were less likely to convert to a negative test (OR = 0.49; 95%C I 0.31-0.78), and HIV positive were more likely to convert to a negative test, although HIV status did not reach statistical significance (OR = 1.50; 95% CI 0.97 - 2.31). The conversion rates by standard smear microscopy during the rest of the treatment period (8, 12 and 20 weeks) were not significantly different between HIV positive and HIV negative patients (Table 3 &4)

**Table 3:** Proportions of negative cases (conversion rates) after different durations of treatment according to HIV status

Type of test	Duration of treatment (weeks)	HIV positive % (n/N)	HIV negative % (n/N)	P value
***Zeihl Neelsen smear***				
	2	72.8(107/147)	63.3(197/311)	0.0457
	8	98.6(139/141)	97.0(295/304)	0.52
	12	99.3(140/141)	98.0(298/304)	0.56
	20	99.3(140/141)	99.0(301/304)	0.80
				
***Fluorescence***	2	72.8(107/147)	63.2(196/310)	0.0433
	8	98.6(139/141)	97.0(294/303)	0.51
	12	99.3(140/141)	98.0(297/303)	0.55
	20	99.3(140/141)	99.0(300/303)	0.80
				
***Culture***	2	59.4(85/143)	51.8(156/301)	0.13
	8	95.7(134/140)	92.5(272/294)	0.21
	12	98.6(137/139)	97.3(285/293)	0.62
	20	100(138/138)	98.6(289/293)	0.40

**Table 4:** Logistic regression analysis of factors* associated with sputum smear** conversion at two weeks of treatment

Variable	OR (95% CI)	P value
Males	0.49 (0.31 - 0.78)	0.002
HIV positive	1.50 (0.97 - 2.31)	0.07

*factors adjusted for were sex, HIV status, age, past history of using anti-TB and education level regardless of their P value

**Ziehl Neelsen and auramine staining gave identical results

OR = odds ratio

CI = Confidence Interval

After two weeks of treatment the conversion rate by fluorescence microscopy was higher in HIV positive (72.8%) than in HIV negative (63.2%) patients by univariate analysis and this difference was also statistically significant (X^2^= 4.08, d.f = 1, P = 0.043). However, when we entered the data in the logistic model and did a backward elimination using stepwise method, variables remaining in the model were sex and HIV status. Males were less likely to convert to a negative test (OR = 0.49; 95% CI 0.31-0.78), and HIV positive were more likely no convert to a negative test, although HIV status did not reach statistical significance (OR = 1.50; 95% CI 0.97 - 2.31). The conversion rates by fluorescence microscopy during the rest of the treatment period (8, 12, and 20 weeks) were not significantly different between HIV positive and HIV negative patients.(Table 3 &4)

With regards to culture, the conversion rate during the whole period of the treatment (2, 8, 12 and 20 weeks) were not significantly different between HIV positive and HIV negative patients.(Table 3)

## Discussion

The study revealed that the conversion rates as judged by standard smear microscopy and fluorescence microscopy after two weeks of treatment was higher in HIV positive pulmonary tuberculosis patients than in those who were HIV negative. However, this applies only to the univariate analysis. After doing a multivariable analysis, the HIV status did not come out as significant anymore. These results are in line with reviews done in past studies, which have indicated that HIV serostatus does not influence sputum smear conversion rate [7,9]. However a study which was done in Karonga district in Malawi showed that the presence of HIV infection was associated with a shorter time to sputum smear conversion [6].

In our study, the conversion rate of culture was not different between HIV positive and HIV negative pulmonary tuberculosis patients. Previous findings such as the one in a study which was done in New Jersey have also shown that HIV serostatus did not influence the rate of documented sputum culture conversion [10]. However our results differ from a study which was done in Spain where it was found that HIV infected patients had a significantly higher culture conversion rate by week 4 than HIV negative patients in the univariate analysis. But, in this same Spanish study after a multivariate analysis, the HIV status did not emerge as being significantly associated with culture conversion during the early period [11].

Currently the duration of infectiousness after the initiation of effective treatment is still a subject of discussion. From our results it is shown that after two weeks of treatment about 30% to 40% of the patients were still potentially infectious, depending on HIV status. This is in contrary to the belief that patients become non-infectious after two weeks of standard treatment regimen. This finding is in line with other results which have also shown that conversion to a negative test and hence the loss of infectiousness of pulmonary tuberculosis patients during therapy does not occur rapidly in all patients [6,11,12]. This finding has implications to those countries which practise patients' isolation during the infectious period and are using two weeks as a time which usually a patient is considered to become non-infectious. For example in the UK, the National Institute of Clinical Excellence (NICE) guidelines indicate that the isolation of smear-positive tuberculosis patients without risk factors for Multi-Drugs Resistant tuberculosis is generally only required for 2 weeks [13].

The results of standard smear and fluorescence microscopy were almost identical and we are inclined to reason that this might have occurred because there was no blinding between the two tests. But it may also reflect that the quality of microscopy in general was very high in that particular laboratory (CT RL). The HIV sero-prevalence of 33.7% found in our study population is almost twice that of blood donors in the study region (17.4% in 2005) [14]. But this is low compared to the HIV prevalence which has been estimated by the MoHSW which sets the HIV prevalence to about 50% of all tuberculosis patients in Tanzania [2,15]. The reason why the HIV sero-prevalence that we found is low compared to that of the MoHSW might be that we only included smear positive pulmonary tuberculosis. Since most of the HIV positive pulmonary tuberculosis patients are smear negative, it is possible that our study inclusion criteria might have excluded many HIV positive/smear negative pulmonary tuberculosis patients. These HIV positive/smear negative patients are included in the MoHSW prevalence estimates.

Pulmonary tuberculosis patients who were also HIV positive were older compared to those who were HIV negative. Most of those who were HIV positive were in the age group 26-35 years. These results are consistent with the data from Tanzania National AIDS Control Programme of 2005 which shows that the age group 20-49 years remained the most affected by HIV for both sexes, an observation that has remained consistent for several years since the beginning of the epidemic [14].

The proportion of patients with past history of using anti-tuberculosis drugs was higher in HIV positive than in HIV negative pulmonary tuberculosis patients. This may be due to the fact that tuberculosis preventive therapy is an intervention that has been part of the package of care for people living with HIV. In this tuberculosis preventive therapy, patients with latent infection of *M. tuberculosis *are given Isoniazid in order to prevent progression to active disease [15].

## Conclusion

Conversion rate of standard smear microscopy and fluorescence microscopy did not differ between HIV positive and HIV negative pulmonary tuberculosis patients. Also the conversion rates of culture were not different between HIV positive and HIV negative pulmonary tuberculosis patients.

After two weeks of treatment 30% to 40% of the pulmonary tuberculosis patients were still potentially infectious, depending on HIV status. The age of those who were HIV positive was higher compared to those who were HIV negative. Being HIV positive was associated with a past history using anti-tuberculosis drugs.

## Competing interests

The authors declare that they have no competing interests.

## Authors' contributions

All authors contributed to the paper, all authors conceived the study. MS conducted the study. SGM, and OM supervised the research. MS and SGM analysed the data. All authors helped to conceptualise ideas and interpret the findings. MS prepared the draft, other authors helped to review and finalise the manuscript

## Pre-publication history

The pre-publication history for this paper can be accessed here:

http://www.biomedcentral.com/1471-2334/10/210/prepub
